# Hypertensive intracerebral hemorrhage: Which one should we choose between laser navigation and 3D navigation mold?

**DOI:** 10.3389/fsurg.2023.1040469

**Published:** 2023-02-24

**Authors:** Zhengbo Yuan, Qingbo Wang, Qikai Sun, Chenglong Li, Fengzhen Xiong, Zefu Li

**Affiliations:** ^1^ Department of Neurosurgery, Binzhou Medical University Hospital, Binzhou, China; ^2^ Department of Neurosurgery, Qilu Hospital of Shandong University, Jinan, China

**Keywords:** hypertensive intracerebral hemorrhage, minimally invasive, three dimensional printing, laser navigation, comparison of surgical methods

## Abstract

**Background:**

Hypertensive intracerebral hemorrhage (HICH) is a severe life-threatening disease, and its incidence has gradually increased in recent years. Due to the particularity and diversity of its bleeding sites, the early treatment of hematoma needs to be more meticulous and accurate, and minimally invasive surgery is often one of the measures that are commonly adopted now. The lower hematoma debridement and the navigation template created by 3D printing technology were compared in the external drainage of a hypertensive cerebral hemorrhage. Then the effect and feasibility of the two operations were explicitly evaluated.

**Material and methods:**

We performed a retrospective analysis of all eligible patients with HICH who underwent laser-guided hematoma evacuation or hematoma puncture under 3D-navigated molds at the Affiliated Hospital of Binzhou Medical University from January 2019 to January 2021. A total of 43 patients were treated. Twenty-three patients were treated with laser navigation-guided hematoma evacuation (group A); 20 patients were treated with 3D navigation minimally invasive surgery (group B). A comparative study was conducted between the two groups to evaluate the preoperative and postoperative conditions.

**Results:**

The preoperative preparation time of the laser navigation group was significantly shorter than that of the 3D printing group. The operation time of the 3D printing group was better than that of the laser navigation group (0.73 ± 0.26 h vs. 1.03 ± 0.27 h *P* = 0.00070). In the improvement in the short-term postoperatively, there was no statistically significant difference between the laser navigation group and the 3D printing group (Median hematoma evacuation rate *P* = 0.14); And in the three-month follow-up NIHESS score, there was no significant difference between the two (*P* = 0.82).

**Conclusion:**

Laser-guided hematoma removal is more suitable for emergency operations, with real-time navigation and shortened preoperative preparation time; hematoma puncture under a 3D navigation mold is more personalized and shortens the intraoperative time course. There was no significant difference in therapeutic effect between the two groups.

## Introduction

Hypertensive intracerebral hemorrhage (HICH) is a severe life-threatening disease with high mortality (1), and even patients who do not die will still have severe residual symptoms after systemic treatment (2, 3). HICH is one of the most severe complications of hypertension and is common in middle-aged men. It is caused by poor elasticity and increased fragility of intracranial arterioles caused by long-term hypertension (4, 5). In China, the incidence of stroke has increased significantly (247/100,000), in the past few years; similarly, the mortality rate (115/100,000) is also the highest in the world (6). Early, timely medical intervention is critical for intracranial hemorrhage. So, the rapid development of the best treatment plan is a priority (7). Even though conservative treatment is an option for most patients, immediate external hematoma drainage is necessary for patients with extensive intracerebral hemorrhage (3, 8).

Currently, primary intracerebral hemorrhage (ICH) treatment is still controversial. Previous studies have shown that surgical treatment has a better prognosis for HICH than conservative treatment (*P* < 0.001) (9). Recent reports indicate that surgical methods such as craniotomy, burr hole, urokinase infusion, drainage catheter, and neuroendoscopic surgery are safe and effective (10, 11). With the development of science and technology, more and more cutting-edge technologies are applied to clinical work—3D printing technology, stereotaxic, neuro navigation, and other technical methods are gradually applied to neurosurgery. The 3D printing technology is based on CT data and 3D software modeling of the original Digital Imaging and Communications in Medicine (DICOM). Based on this technology, individualized analysis of the shape and location of the patient's hematoma is carried out, and the surgical approach is designed to avoid essential parts such as the venous sinus, functional area, and frontal sinus (12). The process is based on the patient's facial features, designing a facial model with pilot holes and printing it with a 3D printer. During hematoma puncture, the puncture angle and puncture position can be fixed through the guide hole, reducing potential tissue damage caused by inaccurate positioning and repeated puncture (11). However, these printing equipment and materials are expensive and challenging to popularize in primary medical institutions in developing countries. Therefore, developing an assisted surgical technique with reasonable prices and high accuracy is necessary.

The laser-guided hematoma puncture technology designed and developed by our clinical team has achieved satisfactory results in the auxiliary treatment of brainstem hemorrhage (13) and trigeminal neuralgia (14), and has been applied to the treatment of spontaneous supratentorial intracerebral hemorrhage. Based on ensuring accuracy and safety, laser-guided puncture of cerebral hematoma has the advantages of real-time positioning, low cost, and short preoperative preparation time, and it is more suitable for application in primary hospitals in developing countries. This study aimed to compare the treatment of hypertensive intracerebral hemorrhage under laser navigation and 3D printing-assisted treatment of hypertensive intracerebral hemorrhage. We want to explore further the clinical effects of these two new surgical methods in treating hypertensive intracerebral hemorrhage.

## Materials and methods

### Patient population

A total of 43 patients with HICH were studied retrospectively in our department between January 2019 and January 2022. All patients were diagnosed with Hypertensive intracerebral hemorrhage (HICH) on initial computed tomography (CT) scans, and the intracerebral hemorrhage (ICH) volume was calculated by 3D Slicer Software (http://www.slicer.org). All patients had apparent symptoms of neurological dysfunction and were indicated for surgery. These patients were divided into two groups: The laser navigation group (Group A, *n* = 23) 3D printing group (Group B, *n* = 20).

All the patients were diagnosed with craniocerebral CT and contrast-enhanced CT. Both examinations were used to rule out arteriovenous brain malformation and 3D reconstruction. The hematoma volumes (HV) were estimated by the 3D printing software Mimics 16.0. Each patient was initially admitted to the neurosurgical ICU and given preoperative preparation. Systolic blood pressure (SBP) was controlled and maintained between 100 and 120 mmHg with an anesthesiologist's assistance.

### Inclusion and exclusion criteria

Inclusion criteria for the patients in this study were as follows: diagnosed with HICH (the diagnostic criteria refer to the relevant recommendations in the *Chinese Multidisciplinary Diagnosis and Treatment Guidelines for Hypertensive Intracerebral Hemorrhage*); CT scan showed hemorrhage from the subcortex, basal ganglia, internal capsule or thalamus(mostly spontaneous supratentorial intracerebral hemorrhage), with or without intraventricular extension, and CT scan showed markedly increased Intracranial Pressure (ICP) performance (shift of midline structure more than 5 mm; compression of ipsilateral ventricle more than 1/2; an ipsilateral cistern, sulcus blurred or disappeared; Glasgow Coma Scale (GCS) scores ≥5; stable vital signs.

Exclusion criteria: the intracerebral hemorrhage was caused by secondary factors (e.g., arteriovenous malformation; aneurysm; tumor stroke; head injury); Hemorrhage in the cerebellum, subcortex, and brain stem; no obvious clinical symptoms and imaging manifestations of intracranial hypertension; GCS < 5; multiple intracranial hemorrhages; severe visceral disease or clotting disorders.

### Surgical management

The ethics committee approved this study at Binzhou Medical University Hospital. All methods were performed following the relevant guidelines and regulations, and informed consent was obtained from all the subjects. All methods of this study were performed under the Guidelines for the Diagnosis and Treatment of Cerebral Hemorrhage in China (2015).

## Surgery

### Hematoma puncture treatment under laser navigation

**Figure 1:**
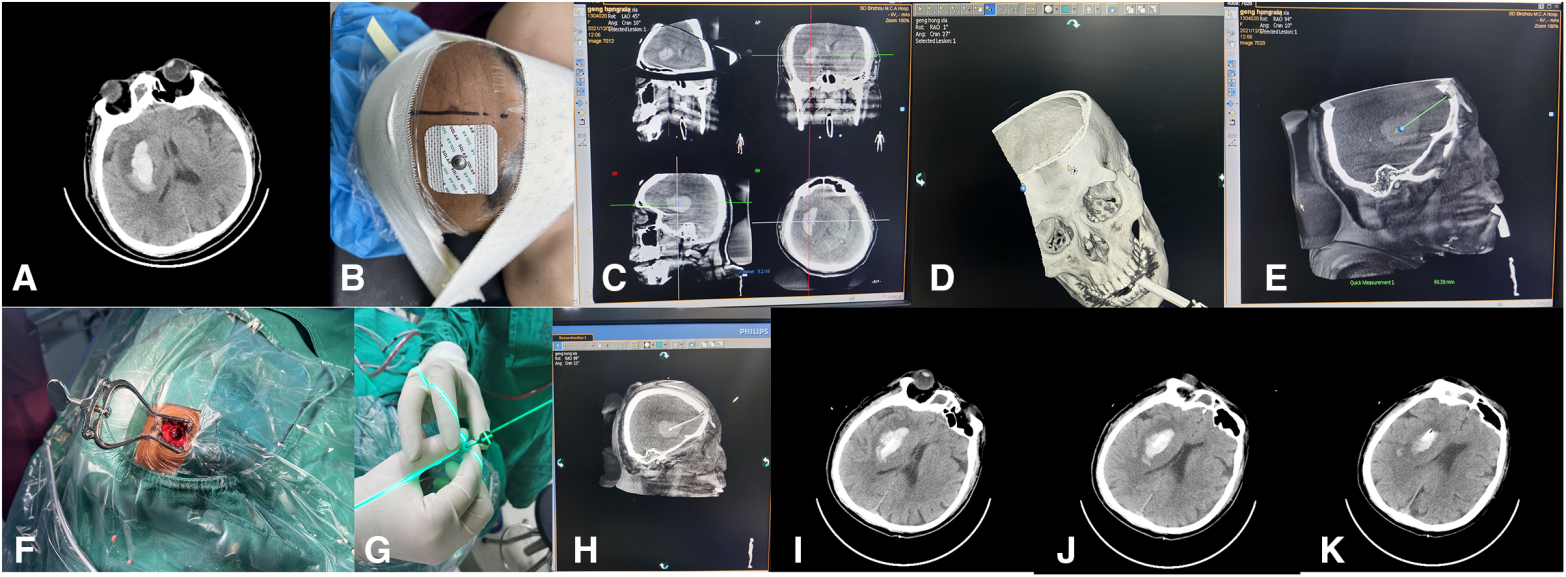
The surgical procedure of laser navigation puncture and drainage to treat basal ganglia hemorrhage.

Before surgery, according to the CT image (Figure 1A), the x-ray-opaque gauze development line was knotted and attached to the forehead scalp to mark the scalp puncture point (Figure 1B). Use the 3D reconstruction software that comes with the DSA machine (Philips Allura Xper FD20 angiography system) to perform Xper-CT scanning, collect raw data for processing and 3D reconstruction, rotate the 3D stereoscopic image, overlap the developing line with the hematoma, and use the principle of linear axiom one to determine. The direction of the laser is emitted, and the working angle of the instant 3D reference image is recorded. The 3D image is cut according to the marked point, the position of the patient's hematoma and the puncture channel is finally determined, and the distance from the scalp to the center of the hematoma is measured (Figure 1C,D). With the puncture point as the center, a 3 cm long transverse incision was made in the forehead hairline. A bone hole is formed, 1 cm in diameter (Figure 1F). Xper-CT scan again selects the skull puncture hole and the long axis of the hematoma as the puncture direction and uses the 3D-APC function in the Xper-APC automatic position control module to position the gantry movement to the angle displayed by the 3D reference image. Attach the bottom end of the laser transmitter to the FD plate so the laser is perpendicular to the FD plate, move the laser transmitter, and align the cross-laser focus to the puncture point (Figure 1G). At this time, the laser emission direction will pass through the puncture point and the hematoma simultaneously for the puncture direction. During the puncture process, the laser focus was continuously placed in the center of the needle tail, and the puncture direction of the trocar remained continuously towards the direction of the hematoma. Fix the laser system, use a 12-F extraventricular drainage tube with the aid of laser navigation, puncture the drainage tube into the center of the hematoma cavity, stop the needle insertion when the puncture is the same as the measured distance, and pull out the needle core when the bloody fluid flows out of the tube. The drainage tube was pulled out, fixed through the subcutaneous tunnel, and connected with the external drainage bag. Then a CT examination was performed to confirm the flexible catheter's positioning and the residual hematoma's stability (Figure 1H–K). Finally, the skin is sutured layer by layer. The hematoma was continuously liquefied with urokinase (20,000U–40,000U urokinase/2–3 ml saline solution) for 2 to 4 days.

### 3D-printed model-guided hematoma puncture treatment

**Figure 2:**
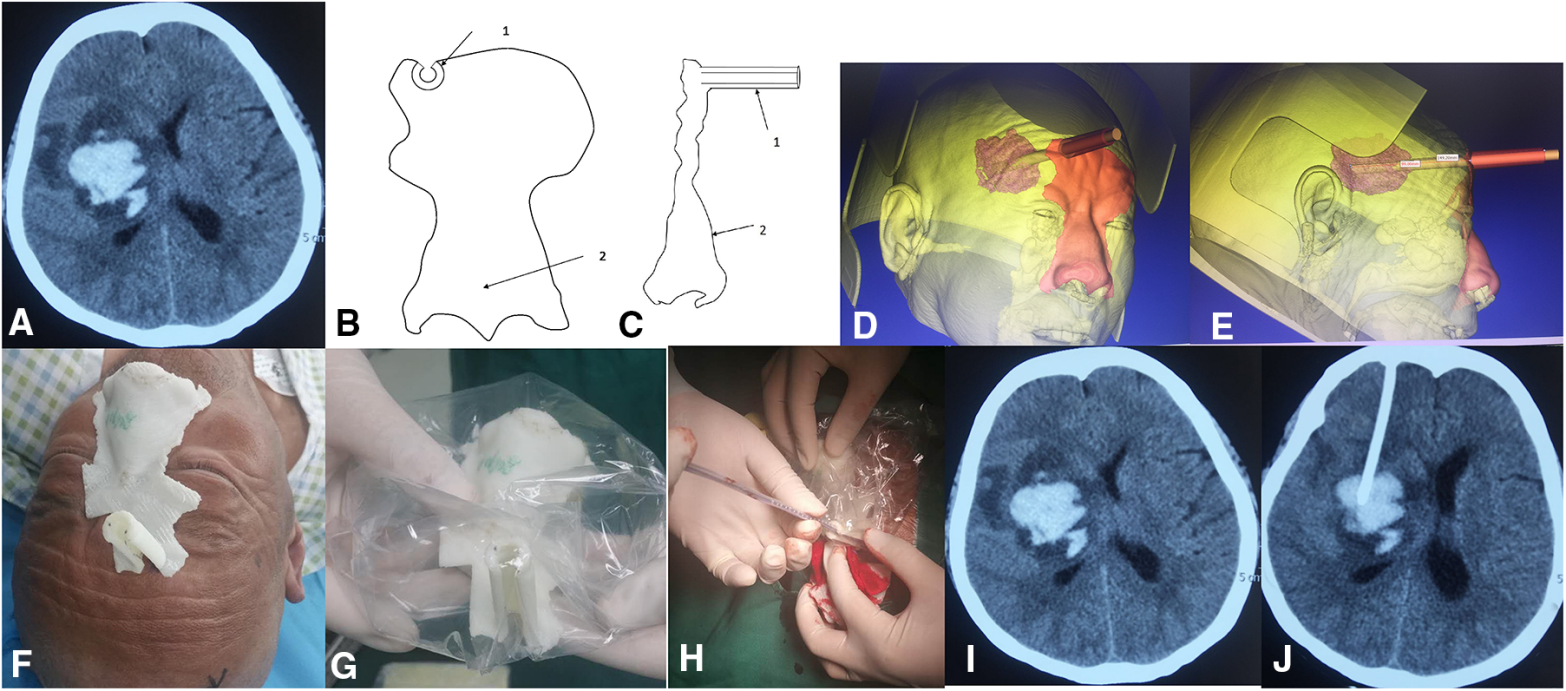
The 3D printing model guides the surgical method for hematoma puncture treatment.

On admission, all patients underwent brain CT scans (Figure 2A). The DICOM files of the CT images were imported into the 3D printing software Mimics 16.0 for 3D reconstruction. Three-dimensional reconstructed images of the hematoma and the patient's scalp were superimposed on a digital computer. The system uses computer-aided design to determine the appropriate entry point, trajectory into the hematoma, and depth of endoscopic insertion (Figure 2B,C). The average scan and reconstruction time was up to 15 min. On the 3D-reconstructed CT images, the hematoma center was superimposed on the scalp through the anterior-posterior (AP) axis, and then the surgical entry point was determined. We used the sinuses as a reference point to determine the frontal entry point. The distance from the sinuses to the directly anterior entry point was measured on the 3D reconstructed CT images. The distance from the frontal entry point to the center of the hematoma was calculated from the 3D reconstructed CT images. Then, a 3D model of the face with extracranial pilot holes was constructed using the 3D image file of the head and printed using a 3D printer (CASET 250MC, China). In our hospital, polylactic acid (PLA) was used as the raw material for printing using the Fused Filament Fabrication (FFF) technique (Figure 2D,E). The 3D-printed facial model is further customized for each patient. The inner surface of the face model is trimmed until a precise fit to the patient's face is achieved without rotation or displacement of the model after fixation. The model is then aseptically processed in preparation for surgery (Figure 2F,G).

With the patient in the supine position, the 3D personalized model is placed near the patient's face to ensure accurate positioning of the bridge of the nose and the zygomatic arch. Next, we place a marker on the patient's frontal puncture site along the channel. The surgical area is then routinely disinfected. The entry point is under the extracranial guide hole of the 3D facial model. The guide hole of the model is used to determine the required drilling position and direction of the puncture needle. The 3D-printed model mask is fixed, and the 12F extraventricular drainage tube is inserted into the bone drilled by the bone drill. The hole penetrates the dura mater in the direction of the puncture channel until the desired depth (the length of the airway plus the intracranial length) is reached (Figure 2H). When the dark red liquid begins to flow, stop the advancement of the drainage tube, pull out the needle core, and the drainage tube passes subcutaneously. The tunnel is pulled out, fixed, and connected to the external drainage bag. The skin is sutured layer by layer, and a CT scan was performed promptly 6 h after surgery (Figure 2I,J). The hematoma was continuously liquefied with urokinase (20,000U–40,000U urokinase/2–3 ml saline solution) for 2 to 4 days.

### Imaging and clinical evaluation

Postoperative CT is performed within six hours after surgery to determine the effect of surgery. The hematoma evacuation rate (ER) was defined as 100−(postoperative volume)/(preoperative volume) × 100%. The following parameters were included for intraoperative efficacy: preoperative preparation time and operation time. Short-term and long-term efficacy evaluation indicators after surgery are (A) surgical complications, such as pneumonia and intracranial infection; (B) length of hospital stay (C) GCS was used to assess the state of consciousness at discharge (D) surgery NIHSS score after three months.

We use the GCS to assess the neurological status in discharge. Postoperative follow-up was performed three months postoperatively, and the National Institute of Health Stroke Scale (NIHSS) scale was employed for clinical efficacy evaluation.

## Statistical analysis

GraphPad Prism 9.0.0 was used in this study, and the measurement data conforming to a normal distribution are expressed as the mean ± standard deviation (mean ± SD). The significance level was set to 5%. Demographic and clinical characteristics of the participants were compared between groups upon admission using Student's *t*-test, when *P* < 0.05, indicating a statistically significant difference. Whereas categorical data were compared using *χ*^2^ tests. The Wilcoxon Rank Sum Test was used to examine the GCS score at admission, the GCS at discharge, the GCS of improvement, and the NIHSS score three months after surgery, and the result is represented by Median (Interquartile Range).

## Results

**Figure 3:**
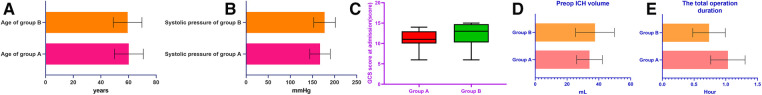
The histograms in the figure represent essential characteristics such as age, systolic blood pressure on admission, GCS score on admission, and preoperative hematoma volume, as well as the comparison results of the average operative time between the two groups, respectively.

There were no significant differences in essential characteristics such as age, systolic blood pressure on admission, GCS score at admission, and preoperative hematoma volume between the two groups (*P* < 0.05) (Table 1) (Figure 3A–D). There were 23 patients in group A, including 13 males and ten females, ranging in age from 41 to 80 years old, with an average age of 60.2 ± 10.5 years. The median preoperative GCS score was 11, and the preoperative hematoma volume ranged from 20 to 51 ml (mean, 34.12 ± 8.08 ml). There were 20 patients in group B, 12 males and eight females, aged 37–74 years, with an average age of 59.4 ± 10.4 years. The median preoperative GCS score was 12, and the preoperative hematoma volume was 20 to 59 ml (mean, 37.60 ± 12.11 ml). There was no significant difference in the preoperative hematoma volume between the two groups (*P* = 0.27).

**Table 1:** General information of the 43 HICH patients.

	Group A (*n* = 23)	Group B (*n* = 20)	*P*-value
Age, year	60.2 ± 10.5	59.4 ± 10.4	0.79
Systolic pressure on admission, mmHg	167.2 ± 23.1	177.5 ± 24.4	0.16
Sex ratio (M:F)	13:10	12:8	0.090
GCS score at admission [*M* (*IQR*), score]	11 (2.5)	12 (3.5)	0.26
Preop ICH volume, ml	34.12 ± 8.08	37.60 ± 12.11	0.27

Values were reported as the mean ± standard deviation (range). The GCS score at admission is represented by [M (IQR), score] (M: Median; IQR: Interquartile Range).

Group A, Laser navigation group; Group B, 3D printing group; GCS, Glasgow Coma Scale; ICH, Intracerebral hemorrhage; M, Male; F, Female; Preop, Preoperation; HICH, Hypertensive intracerebral hemorrhage.

All operations were completed according to consensus specifications, and the average operation time of the two groups was compared (Table 2). The operation time of group A was 0.53–1,50 h, with an average of 1.03 ± 0.27 h. The operation time in group B was 0.33–1.08 h, with an average of 0.73 ± 0.26 h. There was a statistically significant difference in the average operation time between the two groups (*P* < 0.01) (Figure 3E).

**Table 2:** Operative result of 43 patients with HICH.

	Group A (*n* = 23)	Group B (*n* = 20)	*P*-value
The preparation time of 3D model (3D modeling and printing), h		1.28 ± 0.04	
The total operation duration, h	1.03 ± 0.27	0.73 ± 0.26	0.00070*
Postop ICH volume, ml	28.24 ± 8.50	28.33 ± 13.44	0.98
Median hematoma evacuation rate, %	17.67	25.54	0.14
Pneumonia rate, %	4 (17.4%)	3 (15.0)	
Postoperative hospitalization, days	28.74 ± 17.26	21.35 ± 7.99	0.080
GCS score improvement [*M* (*IQR*), score]	3 (3)	1 (3)	0.060
GCS score at discharge [M (IQR), score]	11 (3.0)	13 (4.5)	0.11
NIHSS score three months after surgery [*M* (*IQR*), score]	2 (5.25)	2 (5.50)	0.82
Mortality rate, %	1 (4.4%)	0 (0%)	

Values were reported as the mean ± standard deviation (range). The GCS score improvement, GCS score at discharge and NIHSS score 3 months after surgery are represented by [M (IQR), score] (M: Median; IQR: Interquartile Range).

Compared with the control group.

Group A, Laser navigation group; Group B, 3D printing group; GCS, Glasgow Coma Scale; NIHSS, National Institute of Health Stroke Scale; Postop, Postoperation; ICH, Intracerebral hemorrhage; HICH, Hypertensive intracerebral hemorrhage.

* *P* < 0.05.

**Figure 4:**
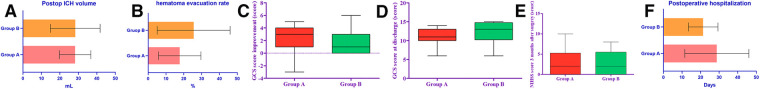
The histograms in the figure represent respectively the comparison of postoperative hematoma clearance rate, GCS score improvement, NIHSS score at 3-month postoperative follow-up, and postoperative hospital stay in groups A and B.

The residual hematoma volume measured by postoperative CT was 28.24 ± 8.50 ml in group A and 28.33 ± 13.44 ml in group B, and the hematoma clearance rates were 17.67% in group A and 25.54% in group B, respectively, and there were no statistics on hematoma clearance between the two groups. The difference was not statistically significant (*P* = 0.14) (Table 2) (Figure 4A,B).

The Median GCS score of group A at discharge was 11, and the Median GCS improvement was 3. The Median GCS score of group B at discharge was 13 and the Median GCS improvement of 1. The GCS scores of the two groups were significantly improved after surgery, and there was no statistical difference between the GCS scores improvement (*P* = 0.060) (Figure 4C,D). There was no significant difference between the NIHSS scores at the 3-month follow-up (*P* = 0.82) (Table 2) (Figure 4E).

The postoperative hospital stay in group A was 28.74 ± 17.26 days (patients who died were excluded); the postoperative hospital stay in group B was 21.35 ± 7.99 days. There was no significant difference in postoperative hospital stay between the two groups (*P* = 0.080) (Table 2) (Figure 4F). One patient in group A died, with a case fatality rate of 4.4%. The patient died of postoperative rebleeding, and no patient died of severe pneumonia or other complications. 0 patients in group B died. Pneumonia is another serious complication that may affect patient outcomes. There were 4 cases of new pneumonia after the operation in group A, and there were no deaths. In group B, there were three new cases of pneumonia after the operation, and no one died (Table 2).

## Discussion

As the elderly population increases yearly, stroke patients are also increasing. There are approximately 2 million new stroke cases in China annually, with an incidence rate of 116 to 219 per 100,000 people (15). Among them, strokes caused by spontaneous intracerebral hemorrhage account for 10% to 20% of all strokes. The morbidity, mortality, and morbidity of hemorrhagic stroke are significantly higher than that of ischemic stroke (16). Many factors are associated with hemorrhagic stroke, including genetics, age, hypertension, diabetes, obesity (16, 17). More and more investigations in China confirm stroke due to high blood pressure (18, 19).

So far, treating hemorrhagic stroke remains controversial, especially the hematoma above the tentorial cerebellar hemorrhage (20, 21). However, an increasing number of studies aim to investigate the efficacy of the early surgical intervention, which can rapidly reduce hematoma volume, relieve hematoma compression on adjacent brain tissue, reduce intracranial pressure, and possibly reduce secondary bleeding caused by hemorrhage-sexual injury (21, 22). Undoubtedly, large, life-threatening hematomas should be surgically removed (23), the second most common non-traumatic brain emergency surgery currently performed in neurosurgery hospitals in most countries (24, 25).

However, traditional craniotomy for hematoma removal is an invasive procedure that fails to protect the still functional brain tissue surrounding the hematoma, causing too much damage (26). Minimally invasive surgery (MIS) includes stereotactic placement of drainage tubes and endoscopic surgery, which has the advantage of less surgical damage and is widely used in treating cerebral hemorrhage. Minimally invasive, simple, and with the shortest duration of surgery, it has the advantages of performing local anesthesia surgery at the bedside in emergencies; the soft catheter avoids mechanical damage to the brain tissue; it is especially suitable for hemorrhagic stroke with moderate hematoma and other advantages (27).

This study compared the effects of two emerging methods of external drainage with catheter placement. The results suggest that the intraoperative duration of puncture surgery assisted by a 3D printing navigation model and the intraoperative duration of laser navigation-guided hematoma debridement are statistically significant. The difference is that the 3D printing group has a shorter intraoperative time, but this difference is based on the preparation of the 3D personalized model for a long time before surgery. If it is necessary to respond to emergency surgery as soon as possible, the 3D printing navigation model is not suitable. There was no significant difference in terms of surgical effect (hematoma clearance rate), and there was no statistical difference in short-term prognosis (GCS improvement) and long-term prognosis (NIHSS score three months after surgery) among all the patients analyzed. However, for the surgery guided by laser navigation, the preoperative preparation time is significantly better than that of the 3D printing group, which has a significant advantage in the case of emergency surgery; moreover, the laser navigation technology is performed during the operation by the members of the surgical team. Real-time navigation and dynamic adjustment are realized based on the cooperation between the two parts. Even if various unpredictable situations cause deviations during the operation, the puncture direction can be adjusted in real-time under laser navigation to achieve flexibility and The perfect unity of precision.

In the past literature, 3D printing technology has been more and more closely integrated with medicine, and can be used as a basic medical technology to combine with different types of clinical work. Its plasticity, diversity and innovation make 3D printing technology have great potential in the medical field (28). In recent years, 3D-Printed Navigation Technology Combined with Neuroendoscopy for Intracerebral Hemorrhage (29), 3D-Printed Titanium Cranioplasty (30), in addition, good results have been achieved in digital anatomy and model making (31). 3D printing mask navigation technology has been widely used in neurosurgery. Compared with traditional small bone window craniotomy, 3D printing mask navigation technology has the advantages of more personalized and accurate. Compared with intraoperative positioning AIDS such as stereotaxic, navigation and robotics, the price advantage of 3D printing technology is often one of the choices of many medical institutions (32).

At the same time, although the surgery assisted by the 3D printing guidance model can achieve a truly personalized treatment plan, this puts forward higher requirements for front-line clinicians, not only to be proficient in the use of modeling-related software, And the tasks of modeling and printing must be completed in a short time. Even with these quick completions, the sterilization of navigation masks remains challenging due to the specificity of 3D printing materials (33), There has yet to be a published normative consensus to regulate the sterilization standard of 3D-printed navigation masks. What makes patients and their families more concerned is the additional cost of 3D printing navigation masks. A study from the University Paris-Saclay on the cost assessment of the use of 3D printing in surgical operations showed that (34): In terms of printing material costs, labor costs, and operating room costs, navigation supplies using 3D printing could eventually add up to several thousand dollars, which can eventually add up to thousands of US dollars, which are still unaffordable for many primary hospitals and the patients who need them.

However, laser navigation can spend less time and workforce on preoperative preparation, saving precious time for rescue; its equipment cost is repeatedly used, there are no consumable raw materials, and the equipment cost is purchased at once. The price is about 15 US dollars (about 100 RMB), the service cycle (under the average working load of the equipment) is about three years or more, and the application of this type of equipment in the medical field has been based for some time (35). The accuracy is very mature, so it is more suitable for promotion and use, especially in economically underdeveloped areas; it has considerable potential general application value.

## Conclusion

The two external drainage tube placement methods have certain advantages in the surgical process; 3D printing navigation model-assisted puncture surgery is a more personalized and advanced surgical method that conforms to the current medical concept. The operation method significantly shortens the intraoperative time based on ensuring accuracy. The hematoma puncture treatment under laser navigation is a low-cost method independently designed and applied by our medical center based on years of clinical experience and practice. Compared with 3D printing navigation model-assisted puncture surgery, the new surgical method with the high cost and operability significantly saves the preparation time before surgery and precious time for emergency and necessary surgery based on ensuring accuracy, and its operation is simple and easy to understand. From the perspective of practicability and popularity, the real-time, accurate, and low-cost method of laser navigation is a more valuable-popularized surgical procedure.

## Data Availability

The original contributions presented in the study are included in the article/Supplementary Material, further inquiries can be directed to the corresponding author/s.
